# Book Review

**DOI:** 10.1155/S146392468000034X

**Published:** 1980


### Laboratory Organisation and Management

*F. Grover and P. Wallace*
*Butterworths Ltd., 1979, pp 241, £5.95, ISBN 408707933*

The authors of this useful book are experienced in laboratory management and safety aspects of medical laboratories and, although their specialism is to some extent reflected in the selection of material, most of the content is of general applicability. There are seven main chapters, Laboratory Planning and Layout, Selection and Management of Staff, Purchasing and Financial Control, Management of Stores, Laboratory Administration, Service Departments and Special Purpose Rooms, and Health and Safety. In addition there are brief chapters on Maintenance of Laboratory Premises and Equipment, Automation in the Laboratory and Management Techniques and Functions. Automation, including the use of computers, is covered in a mere six pages, and although most of the managerial and organizational problems are alluded to, the treatment is inevitably superficial and of rather limited value. There remains a real need for an in-depth evaluation of this complex and rapidly expanding subject area.

The book is intended to provide practical guidance and this indeed is its virtue. The responsibilities of a laboratory manager and the flexibility of approach accorded to him vary widely with the nature and size of the organization he serves. For example, laboratory managers in a large organization are unlikely, however much they might wish to do so, to be able to react to the content of the chapters on staff selection and purchasing and financial control; procedures for such matters will be enshrined in the protocols of the establishment and their amenability to modification, at least in the short term, will be minimal. On the other hand the chapter on health and safety aspects should prove useful to all laboratory managers.

Although primarily intended for laboratory managers, the book has a potentially wider readership. No young scientist intent on a laboratory career should embark on it in ignorance of the essential organizational procedures which exist to maintain a safe and efficient working environment, and a few hours with this book will be time well spent.

At current prices the book is good value, the style is straight forward and pleasantly free from the loftiness which so often attends those works exhorting sound and sensible behaviour.

*J.K. Foreman*

### Product News

### Titration systems

Radiometer have announced the introduction of the DTS885 and DTS886 white liquor titration systems for the automatic analysis of white, green and black liquor. The DTS885 system is for single sample determinations and the other system for on-line determinations. The titration process is microcomputer-controlled with the automatic addition of formaldehyde during the titration. All three equivalence points are automatically printed-out together with the calculated amounts of effective, active and total alkali as well as percent causticity and percent sulphidity. The range of analysis is 30-190 g NaOH/1 total alkali, with a repeatability of 0.4g NaOH/1 total alkali.

*Radiometer A/S, Emdrupvej 72, DK-2400 Copenhagen NV, Denmark.*

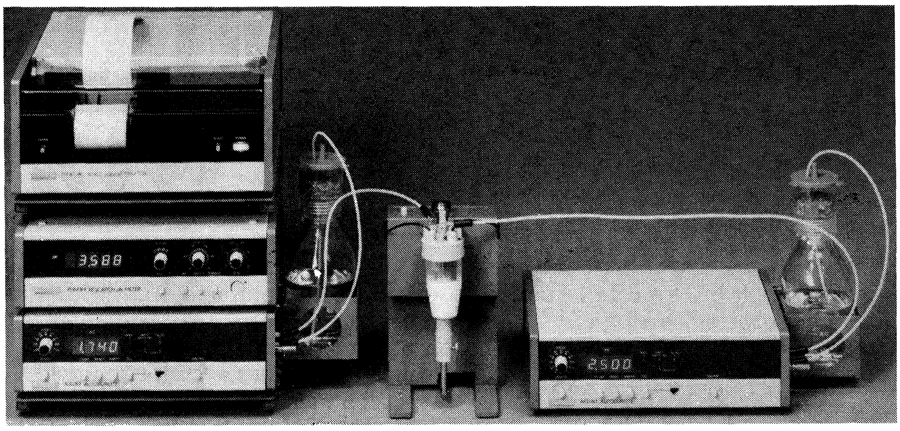

*White liquor automatic titration system from Radiometer*

### Fluxer for sample preparation

The Claisse Fluxer VI is an instrument to make fusions in the most effective way possible. It also makes casting and, depending on the container for casting, the end product is a glass disk if the glass is poured into a mould; or a solution if the glass is poured into a beaker containing an acid solution, following the method of Crow and Connolly. The design has been modified slightly so that one kind of Fluxers can make glass disks and another kind of Fluxers can make glass disks and solutions. In the latter, changing from one function to the other is done by moving one part only from one slot to another slot. It is even possible to make solutions and glass disks in the same time. For easy access to holders and samples at the rear of the instrument, the column can be rotated; it locks at one position for processing.

The fluxer can make any size of glass disks up to about 40 mm diameter; the size of the disks depends on the size of the moulds used.

*Claisse Scientific Corp, 7 Peace Jardins Merici Suite 1301, Quebec, G154N8 Canada.*

